# Bacterial quorum sensing orchestrates longitudinal interactions to shape microbiota assembly

**DOI:** 10.1186/s40168-023-01699-4

**Published:** 2023-11-06

**Authors:** Ying Su, Ming-ying Xu, Ying Cui, Run-zhi Chen, Li-xiang Xie, Jing-xiang Zhang, Yong-qiu Chen, Tao Ding

**Affiliations:** 1 Department of Immunology and Microbiology, Zhongshan School of Medicine, Sun Yat-Sen University, Guangzhou, 510080 China; 2 Key Laboratory of Tropical Diseases Control (Sun Yat-Sen University), Ministry of Education, Guangzhou, 510080 China; 3 Department of Immunology and Pathogenic Biology, Zhaoqing Medical College, Zhaoqing, 526020 China

**Keywords:** Microbiota assembly, Bacterial interaction, Quorum sensing, Interspecies cross-talk, Bacterial communication, Microbiota manipulation

## Abstract

**Background:**

The mechanism of microbiota assembly is one of the main problems in microbiome research, which is also the primary theoretical basis for precise manipulation of microbial communities. Bacterial quorum sensing (QS), as the most common means for bacteria to exchange information and interactions, is characterized by universality, specificity, and regulatory power, which therefore may influence the assembly processes of human microbiota. However, the regulating role of QS in microbiota assembly is rarely reported. In this study, we developed an optimized in vitro oral biofilm microbiota assembling (OBMA) model to simulate the time-series assembly of oral biofilm microbiota (OBM), by which to excavate the QS network and its regulating power in the process.

**Results:**

By using the optimized OBMA model, we were able to restore the assembly process of OBM and generate time-series OBM metagenomes of each day. We discovered a total of 2291 QS protein homologues related to 21 QS pathways. Most of these pathways were newly reported and sequentially enriched during OBM assembling. These QS pathways formed a comprehensive longitudinal QS network that included successively enriched QS hubs, such as *Streptococcus*, *Veillonella*-*Megasphaera* group, and *Prevotella*-*Fusobacteria* group, for information delivery. Bidirectional cross-talk among the QS hubs was found to play critical role in the directional turnover of microbiota structure, which in turn, influenced the assembly process. Subsequent QS-interfering experiments accurately predicted and experimentally verified the directional shaping power of the longitudinal QS network in the assembly process. As a result, the QS-interfered OBM exhibited delayed and fragile maturity with prolonged membership of *Streptococcus* and impeded membership of *Prevotella* and *Fusobacterium*.

**Conclusion:**

Our results revealed an unprecedented longitudinal QS network during OBM assembly and experimentally verified its power in predicting and manipulating the assembling process. Our work provides a new perspective to uncover underlying mechanism in natural complex microbiota assembling and a theoretical basis for ultimately precisely manipulating human microbiota through intervention in the QS network.

Video Abstract

**Supplementary Information:**

The online version contains supplementary material available at 10.1186/s40168-023-01699-4.

## Introduction

Human microbiomes have been empirically revealed to undergo nonrandom and repeatable community assembly and succession, such as the trait-based community assembly revealed in infant gut [1, 2]; as well as the quick recoverable assembly of human oral biofilm microbiome (OBM) after clinical scaling [3]. Mechanisms underlying microbiota assembly are one of the main problems in microbiome research, which is also the primary theoretical basis for precise manipulation of microbial communities [4, 5]. Until now, several theories, such as priority effects [6], metabolic cooperation [7–9], interspecies bacterial competition [10], and hydrodynamic disturbance [11], have been proposed to uncover rules underlying community assembly from the perspective of metabolic interaction. However, metabolism in bacterial flora is extremely complex and redundant, so it is a big challenge to map the metabolic network for precise manipulation on both community structure and function [4]. In this case, excavating an applicable ecological network with more universality, specificity, and regulatory power is of great significance for understanding assembly rules and realizing precise manipulation of bacterial flora.

Bacterial quorum sensing (QS), as the most common means for bacteria to exchange information and interact, is characterized by universality, specificity, and regulatory power and potentially influences the assembly processes of human microbiota. QS has been widely found from human microbiota such as oral cavity [12, 13], skin [14], lung [15], and gut [16, 17]. Among all the above flora, the assembly of OBM is more likely to be regulated by QS due to its compact spatial structure [18, 19] and the hydrophobic glycoproteins coat [20, 21], which promote a higher transmission efficiency of QS signals within it [22]. Combined with its fast and repeatable assembly characteristics [3], OBM is an ideal target to study the role of QS in controlling microbiome assembly.

Due to current cultivation limitations, only three kinds of QS pathways have been experimentally identified in OBM, namely autoinducer peptide (AIP)-based QS represented by *Streptococcus* [23, 24], autoinducer-2 (AI-2)-based QS found in *Fusobacterium nucleatum* [25, 26], and recently discovered (acylated homoserine lactone) AHL-based QS pathway [27, 28]. High-throughput sequencing of target microbiome is beneficial to uncover QS pathways in uncultured microbes from human microflora [29]. However, most studies are based on cross-sectional analysis, ignoring the potential changes and evolution of QS regulation, which limits our understanding of the true role of QS in community assembly. This is a key reason why QS manipulation is studied in simple synthetic microbial communities but is difficult to be widely utilized in larger multispecies communities.

To gain a better understanding of the role of bacterial QS in the assembly of microbiota, we developed an in vitro model that mimics the process of OBM assembly. We conducted a thorough screening of QS pathways in OBM and analyzed their patterns of succession during assembly. We also proposed a longitudinal QS communicating network that deciphers the flow of QS signals. Based on this network, we experimentally verified the predictability and feasibility of interfering the QS network to control OBM assembly. These findings reveal an unprecedented QS network that drives microbiota assembly and provide novel perspectives on the precise manipulation of natural, multispecies microbiota.

## Methods

### In vitro modelling of Oral Biofilm Microbiota Assembling (OBMA)

#### Saliva collection

Saliva samples were collected from 12 dentally healthy subjects, avoiding from antibiotic intakes for at least 3 months. Subjects were required to refrain from food or drink 1 h before saliva donation. Saliva samples were pooled together and processed for subsequent two purposes: as inoculating seeds and media component, separately. For the use as inoculating seeds, around 10 ml pooled saliva was centrifuged at 2600*g* for 10 min at 4 ℃ to remove large debris and eukaryotic cells, and the remaining supernatant with oral microbiota was mixed with same volume of 50% glycerol and stored at − 80 ℃ before use. For the use as media component, the rest of pooled saliva was treated with dithiothreitol (2.5 mM final concentration) [30] for 10 min and centrifuged at 17,500*g* for 30 min at 4 ℃. The supernatant was collected, filter-sterilized, and stored at − 20 ℃ before use.

#### Cultivation conditions

**Fig. 1:**
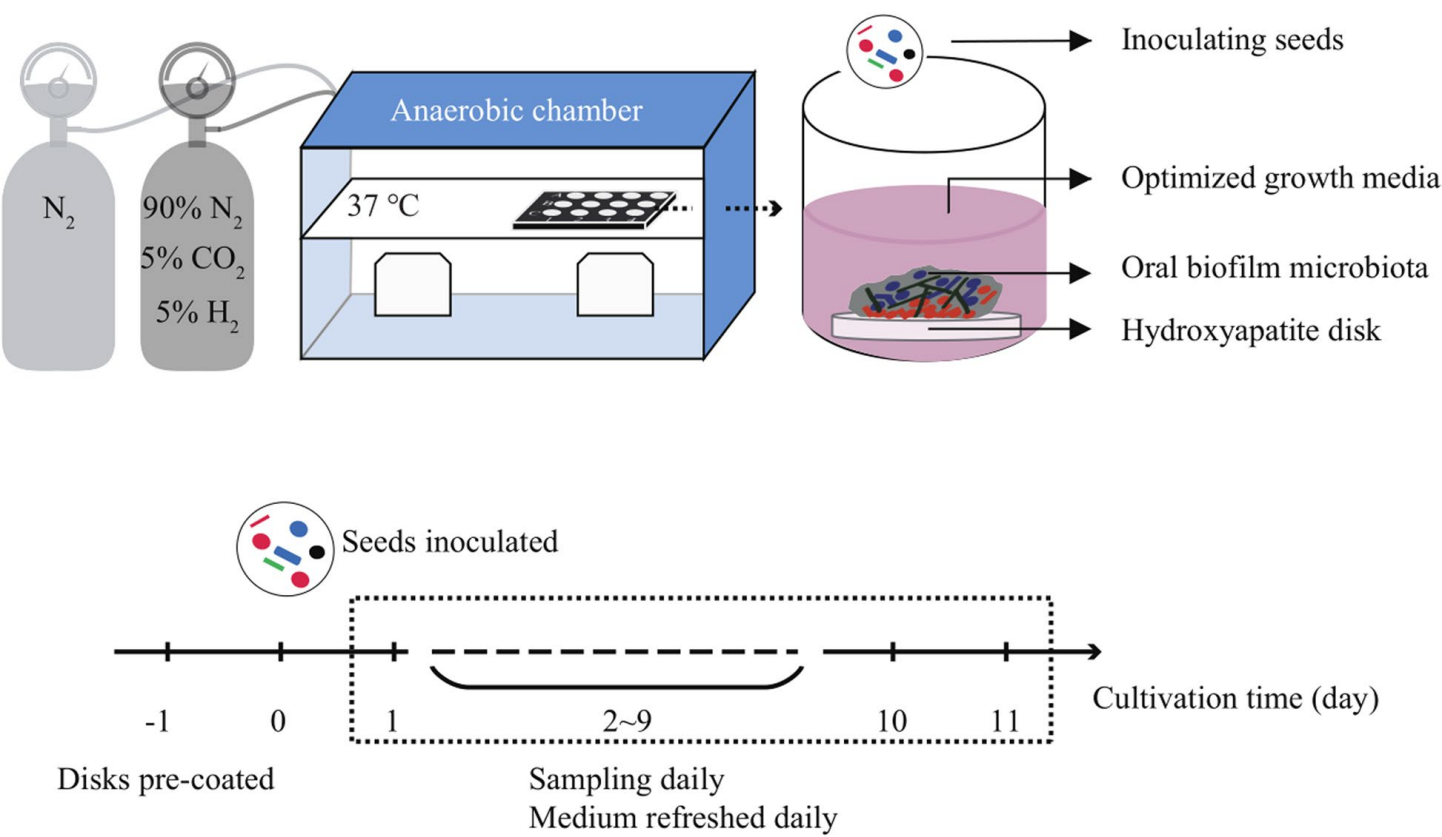
In vitro modelling of oral biofilm microbiota assembly (OBMA) and the study design

The device for oral biofilm microbiota (OBM) assembling consisted of a 12-well polystyrene cell culture plate, optimized growth medium (OGM), and hydroxyapatite (HA) disks as previously described [31] as biofilm growth substrata (Fig. 1). HA discs were pre-coated with sterile saliva 1 day before OBM assembling (day − 1). To initiate assembly of OMB, the model was inoculated with 1% seeds in an optimized growth medium consisting of 30% sterile saliva, 60% modified mFUM (Guggenheim B et al., 2001) (supplemented with 1 mM L-arginine, 10 mg/L *N*-acetylmuramic acid, 1 mg/L hemin, 0.2 mg/L vitamin K, 0.1% sucrose, 0.1% glucose), 10% FBS, and 50 mmol/L PIPIES at pH 7.0 (day 0). Cultures were incubated with 8 replicates at 37 °C for 11 days in an anaerobic chamber (90% N_2_, 5% CO_2_ and 5% H_2_; Don Whitley Scientific Limited, UK) to favor the assembly of OBM. The culture medium was refreshed with OGM daily. Disks without inoculating treatment were conducted as negative controls.

#### Sampling

OBM samples developed on the HA discs were collected daily. Briefly, the collected samples were “dip-washed” three times in sterile phosphate-buffered saline to remove the culturing suspension and the intact biofilm-discs were frozen at 80°C for further DNA extraction with the DNeasy PowerBiofilm Kit (Qiagen).

#### Quantification

Quantitative PCR (qPCR) was performed as previously described [32] to assess the abundance of bacteria in OBM samples. qPCR reactions were performed in triplicate in a 20-µl system including 10 µl of 2 × SYBR Premix ExTaqII (TakaraBioInc), 0.2–0.4 µM of primer sets Eub338F/Eub518R, and 2 µl of 1/10 diluted template DNA. qPCR was performed with the CFX96 Touch (Bio-Rad) using the standard reaction conditions according to the standard operating manual.

### Shotgun sequencing and bioinformatics

The DNA extracted from the OBM samples and control samples was quantified using a Qubit 4.0 Fluorometer (Life Technologies, Grand Island, NY). High-quality DNA samples from OBM were used for subsequent metagenomic sequencing. Sequencing of paired-end libraries was generated using the Next®Ultra™ DNA Library Prep Kit for Illumina ® (New England Biolabs, MA, USA) following the manufacturer’s recommendations. The resulting libraries were then sequenced on one 2 × 150 bp lane of an Illumina NovaSeq 6000 platform.

The raw data was processed using Trimmomatic (v.0.36) to acquire the clean data for subsequent analysis. Clean reads were assembled into contigs using MEGAHIT (v.1.2.9) with default parameters. Open reading frames (ORFs) were predicted based on assembled contigs using metaProdigal (v.2.6.3) with a minimum length of 100 nt. A non-redundant gene catalog was constructed using CD-HIT (v.4.8.1) with thresholds of 95% identity and 90% coverage.

The taxonomic assignment of each unigene was performed based on the result of gene annotation using MEGAN (v.6.21.7) with LCA algorithm. To calculate the relative abundance of each gene, the number of reads assigned to a specific gene was divided by the length of the gene and subsequently compared to the sum of divided read number of all genes using the BWA-MEM program [33].

### Workflow for the retrieval of QS proteins from OBM metagenomes

#### Construction of a reference database of QS synthases and receptors

According to the Sigmol [34] and the Quorum Peps [35] databases, proteins participating in synthesizing or sensing 26 different types of QS signals have been experimentally characterized and summarized into a list of QS systems in a previous study [36]. Protein sequences of the listed QS proteins were obtained from NCBI (https://www.ncbi.nlm.nih.gov/) and UniProt (https://www.uniprot.org/) and were used as training sequences to create a QS reference database.

Putative homologues of QS proteins in OBM metagenomes were retrieved using the BLASTP command implemented in DIAMOND against the QS reference database with the following thresholds: sequence identity ≥ 30%; alignment coverage ≥ 50%; *e*-value ≤ 1e^−5^. All retrieved homologues were further submitted on the Conserved Domain Database (CDD) [37] and the non-reductant protein database (NR) at NCBI. Those sequences without conserved domains (CDs) and annotations similar to reference QS proteins were discarded.

Relative abundance of each QS homologues from OBM metagenomes was calculated by summing up the abundances of all sequences affiliated to the specific QS homologue, which was subsequently divided by the abundance of a single-copy housekeeping gene *recA* to get normalized abundance of each QS homologues [38, 39]. The normalized abundance represented the averaging copies of each homologue in an individual cell. The complete workflow for retrieval of QS proteins from the OBM metagenomes is exhibited in Fig. S1.

### QS-interfering experiment

To assess the power of QS in driving the assembly of OBM, another verification experiment with a control group (3 replicates) and an AI-2 signal interfering group (3 replicates) was conducted using the OBMA model (Fig. S2). All cultures were incubated at 37 °C for 7 days to fulfill a complete assembly of OBM. Differently, the interfering group was additionally supplemented with D-ribose, a frequently used AI-2 inhibitor [40, 41], to interrupt the AI-2 based signaling from day 2 to day 7. The OBM disks were sampled to extract DNA and quantified as previously mentioned. Community structures of the collected OBM samples were analyzed by sequencing 16S rRNA gene amplicons in Magigene (Guangzhou, China). Briefly, the variable region four (V4) of bacteria 16S rRNA gene was amplified using primer set 515F/806R [42] and sequenced on one Illumina Hiseq2500 PE250 platform. All the obtained paired-end reads of 16S rRNA gene amplicons were analyzed using QIIME [43] to obtain their taxonomic assignments and calculate their relative abundances. All libraries were rarefied to an even depth based on the smallest sample.

### Statistical analysis

All statistical analyses were performed in RStudio (v.1.4.1103) with R (v.4.2.1). Shannon index was calculated to characterize microbial diversity using the vegan package [44]. Differences and taxonomy classification in microbial communities were calculated and visualized by a PCA ordination plot and a histogram plot respectively using the MicrobiotaProcess package [45]. Nonparametric Spearman’s correlation test was used to test the associations between genus and annotated metabolic pathways of OBM. Time periods when key genera were differentially abundant between the AI-2 interfering and control group were evaluated using the “MetaDprof” R package with smoothing spline ANOVA (ssANOVA) [46]. All the analyses were unpaired.

## Results

### In vitro OBMA model is robust to simulate the assemble process of OBM

**Fig. 2:**
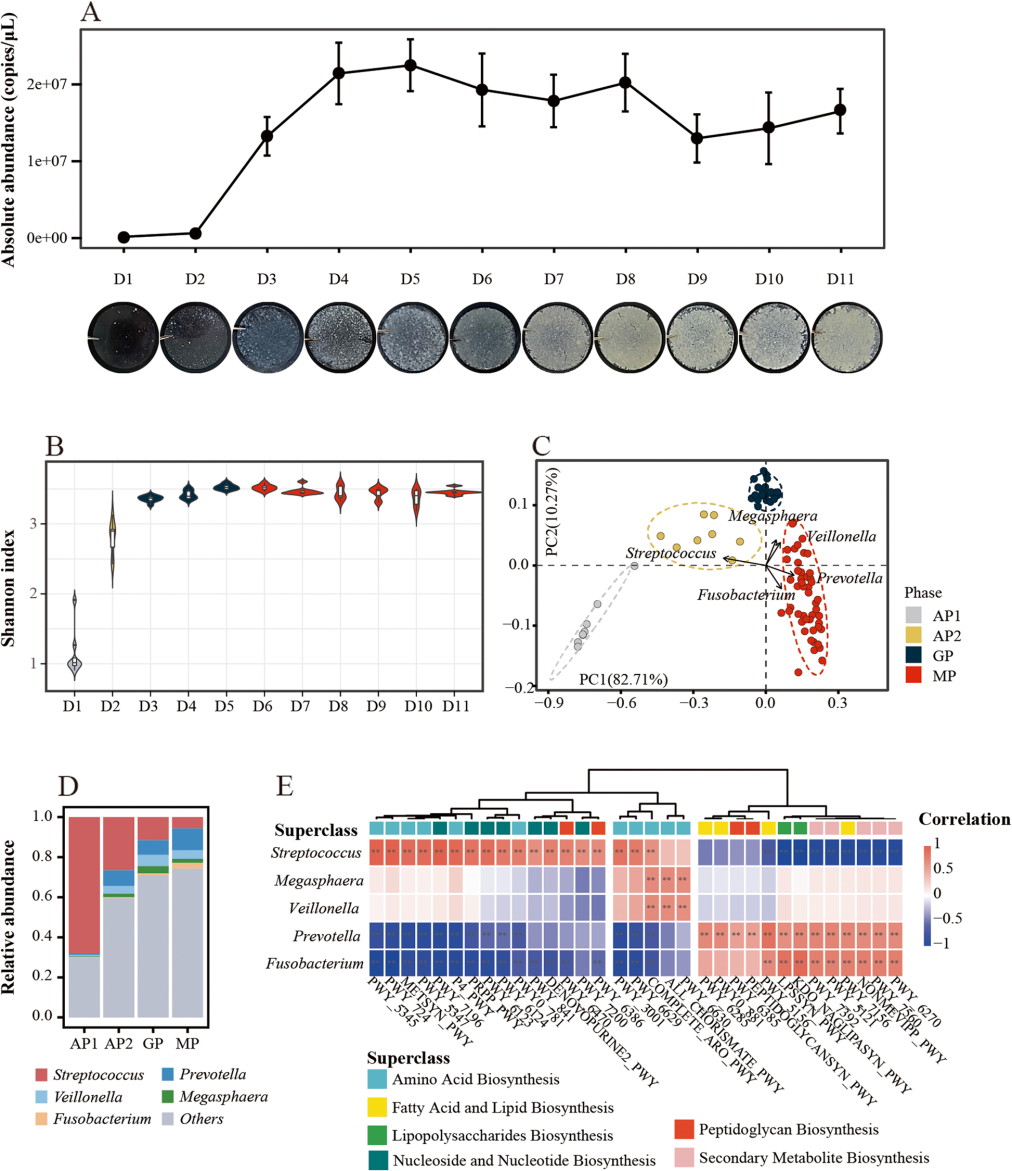
Evaluation of in vitro OBMA model for simulating the assembly of oral biofilm microbiota (OBM). **A** Time-series photographs show the development of OBMs using the OBMA model, reflected by changes in biomass. **B** Alpha diversity of OBMs assembled by the OBMA model over time. **C** Microbiota composition of OBM shown in robust principal component analysis (PCA) biplot, which divided the assembly process into four distinct phases. **D** The succession of top five genera and their associated main functions during OBM assembly. **E** The *x*-axis represents the metabolic pathways annotated in the OBM metagenome. Each column in the figure is labeled with the corresponding BioCyc ID of the specific metabolic pathway. The correlation coefficients are calculated by nonparametric Spearman’s correlation between core genera in OBM and functional pathways

The in vitro OBMA model uses pooled saliva as the inoculum seed and a modified OGM, which can well simulate the naturally complex conditions for OBM assembly. During 11 consecutive days of culture, we observed the complete assembly process of OBM from attachment, expansion to maturation using the OBMA model (Fig. 2A). Based on the successional characteristics of the assembled OBM, we divided the whole process into three periods: the adapting phase (AP) from day 0 to day 2, the growing phase (GP) from day 2 to day 5, and the mature phase (MP) from day 5 to day 11.

During AP, oral bacteria started to colonize with the smallest biomass, which was hardly observed on the surface of the HA discs (Fig. 2A). We noticed that, even no significant difference of biomass in former 2 days, the Shannon index of the OBM on day 2 had a distinct increment compared to that in day 1 (Fig. 2B). This implies that a large and diverse group of bacteria were recruited before rapid development of OBM. In this case, we subdivided the AP phase into AP1 stage (day 0 ~ day 1) and AP2 stage (day 1 ~ day 2). During the period of AP (especially at AP1 stage), *Streptococcus* showed absolute abundance advantage in OBM (Fig. 2C and D; around 30 ~ 70%) and assumed more responsibilities for amino acid and nucleotide biosynthesis (Fig. 2E). After that, the assembly of OBM grew exponentially in GP (Fig. 2A), during which the richness was slightly increased (Fig. 2B). At this stage, the community structure was relatively stable (Fig. 2C) and was mainly characterized by the increment of *Veillonella* and *Megasphaera* (Fig. 2C and D). Once assembled into MP, *Prevotella* and *Fusobacteria* were further enriched (Fig. 2C and D) and the function of MP-OBM demonstrated more mature and invasive features such as peptidoglycan and secondary metabolite biosynthesis (Fig. 2E); we therefore concluded that the complete assembly of OBM was reproduced in vitro using the OBMA model.

The whole assembly process followed the trajectory of “adaptation-rapid proliferation-maturation,” during which the core genus succeed from *Streptococcus*, passing by *Veillonella* and *Megasphaera*, finally to *Prevotella* and *Fusobacterium* (Fig. 2C and D). The assembly process simulated by the OBMA model is consistent with the observed development of human oral plaque in previous studies [3], which reflects the reliability of the OBMA model in simulating the real assembly process and provides a reliable basis for studying the assembly rules and mechanisms of complex multispecies microbiota.

### Diverse QS pathways were identified through mining OBM genomes

In this study, we created a QS reference database, containing 415 reference protein sequences linked to 26 types of QS pathways. This database was used as a subject data in a BLASTP search for homologues of QS proteins in OBM genomes. Using stringent criteria, 7531 putative protein sequences were identified from the non-redundant gene catalog of OBM genomes. After careful screening for correct functional CDs and annotations corresponding to the reference QS proteins, 2291 homologues were identified that were associated with 21 distinct QS signals. Among these, only 10 QS pathways had both signal-synthesizing and signal-sensing proteins (Table 1), while the remaining QS pathways were incomplete with only a signal-synthesizing or signal-sensing protein (Table S1). To ensure the necessity for signal delivery, only the 10 complete QS pathways were selected for further analysis. The roles of these pathways, summarized in Table 1, are mainly involved in regulating metabolisms such as bacteriocin production, biofilm formation, virulence, and more, through intraspecies, interspecies, and even interkingdom communications. Importantly, this study provides the first comprehensive picture of QS-based communicating in OBM.

**Table 1:** Proposed roles for the homologs of reference QS proteins found within the oral biofilm microbiota

**Signal**	**Reference protein**	**Mapped genus (top 5)**			**Possible roles regulated by the QS circuit**
		**AP1**	**GP**	**MP**	
**AP specific**					
**AIP_Agr-Fsr_Like**	AgrD synthase (Streptococcus pneumoniae R6)	Streptococcus	\	\	Virulence (proteases), biofilms (as in Enterococcus faecalis) [47, 48] Competence, biofilms (as in Streptococcus spp) [49, 50] Adhesion, bacteriocin production (Lactobacillus spp.) [51, 52] Virulence (hemolysins, surface proteins) (as in Staphylococcus spp.) [53, 54]
	AgrC receptor (Streptococcus pneumoniae R6)	Streptococcus	Streptococcus	Streptococcus	
**Lantibiotics**	NisA synthase (step 1) (Lactococcus lactis subsp. lactis)	Lactococcus Streptomyces Bacillus	\	\	Antimicrobial peptides [55]; Activation of autoinduction process and its siblings [56] Inter- and intraspecies signaling molecule [56]
	CylM; NisC peptide processor (Lactococcus lactis subsp. lactis)	Streptococcus	Streptococcus	Streptococcus	
	NisK receptor (two components)	Streptococcus Unclassified Firmicutes	Streptococcus Clostridium Peptoniphilus	Streptococcus Unclassified Firmicutes Tepidibacter Peptostreptococcus Unclassified Oscillospiraceae	
**Bacteriocin-II**	CbnS synthase (Carnobacterium maltaromaticum)	Streptococcus	Streptococcus Prevotella	Prevotella	Serving as toxins in interference competition, preventing the invasion of other species, and enabling the producer strain to establish itself in a new community (as in oral Streptococci) [57–59]
	CbnK receptor (two components)	Streptococcus	Streptococcus Mogibacterium	Streptococcus Mogibacterium Unclassified Lachnospiraceae	
**GP specific**					
**AI-2**	Pfs synthase (step 1) (Salmonella enterica); LuxS synthase (step 2) (Bacteroides vulgatus)	Streptococcus	Streptococcus Prevotella	Streptococcus Prevotella Fusobacterium Porphyromonas	Interspecies communication [60, 61] Expression of virulence factors, motility, and biofilm formation [62, 63]
	AibA receptor (Helicobacter pylori)	Streptococcus Veilonella Fusobacterium	Veillonella Streptococcus Fusobacterium	Fusobacterium Veillonella Selenomonas	
	LsrB receptor (Escherichia coli); LuxP receptor (two components) (Vibrio harveyi)	Neobacillus Arthrobacter Serratia	Neobacillus Arthrobacter Klebsiella Unclassified Anaerolineaceae Unclassified Lachnospiraceae	Neobacillus Biomaibacter Arthrobacter Unclassified Lachnospiraceae Enterobacter	
**AIP_RRNPP**	PhrC-Shp-NprX-PapR-CcfA synthase	Streptococcus Methylomusa Neisseria Carnobacterium	Streptococcus Methylomusa Megasphaera Chlamydia Dialister	Methylomusa Streptococcus Megasphaera Fusobacterium Clostridium	Sporulation, conjugation, biofilm formation and pathogenic responses (as in Bacillus and Enterococcus species) [64] Extracellular glucosyltransferase activity required for tooth surface colonization(as in Streptococcus gordonii) [65] Interspecies cross-talking among different streptococci [66, 67]
	Rap-Rgg-NprR-PlcR-PrgX receptor (One Component)	Streptococcus	Streptococcus	Streptococcus	
**DSF**	RpfB synthase (step 1) (Xanthomonas campestris); RpfF synthase (step 2) (Xanthomonas campestris)	Companilactobacillus Unclassified Firmicutes Neisseria Veillonella	Clostridium Veillonella Companilactobacillus Thermoanaerobacterium Prevotella	Veillonella Fusobacterium Companilactobacillus Clostridium Unclassified Odoribacteraceae	Interspecies to interkingdom signaling, regulation of motility, biofilm formation, iron uptake, virulence in other prokaryotes, elicitation of the innate immunity of plants, induction of the stringent response, and siderophore production in other bacteria (as in Burkholderia cenocepacia) [68]
	RpfR receptor (one component)	\	Campylobacter Butyrivibrio Ruminococcus	Butyrivibrio Campylobacter Ruminococcus Selenomonas Desulfobulbus	
	BCAM0227; RpfC; RpfS receptors (two components)	Streptococcus	Prevotella Schaalia Mogibacterium Candidatus Saccharibacteria Bacteroides	Prevotella Bacteroides Mogibacterium Schaalia Fusobacteria	
**HAQ**	PhnA; PhnB; PqsD synthase (Pseudomonas aeruginosa PAO1)	Streptococcus Veillonella	Veillonella Streptococcus Prevotella Unclassified Firmicutes Megasphaera	Prevotella Veillonella Unclassified Firmicutes Eubacterium Fusobacterium	Antimicrobial activity and intercellular signaling [69] Regulating host innate immune responses [70]
	PqsR receptor (two components) (Pseudomonas aeruginosa	Dialister	Dialister Dorea	Dialister Dorea	
**MP specific**					
**AHL**	HdtS; LuxI; AinS synthase	Streptococcus Neisseria	Dialister Acidaminococcus Chlamydia	Acidaminococcus Prevotella Dialister Porphyromonas Fusobacterium	Mediating intra-species, interspecies and interkingdom communication [71] Multispecies biofilm formation [72]
	AinR receptor (two components)	Bacteroides	Bacteroides	Bacteroides	
**AHK**	CqsA synthase	\	Veillonella Prevotella Bacteroides	Prevotella Veillonella Bacteroides Parabacteroides	Bacteria-host interactions, biofilm formation and competence [73]
	CqsS receptor (two components)	Haemophilus Fermentimonas	Prevotella Clostridium Eubacterium Haemophilus Fermentimonas	Prevotella Clostridium Petrimonas Parabacteroides Ruminococcus	
**Ethanolamine**	GlpQ; UgpQ synthase	Enterococcus Vagococcus	Unclassified Eubacteriales Family Bacteroides Fusobacterium Aggregatibacter Chlamydia	Fusobacterium Aggregatibacter Unclassified Eubacteriales Family Bacteroides Clostridium	Expression of genes involved in interkingdom signaling and virulence [74]
	CqsR receptor (two components)	Streptococcus Veillonella	Veillonella Streptococcus Ruminococcus Clostridium Prevotella	Veillonella Fusobacterium Ruminococcus Clostridium Streptococcus	

### The dominant bacteria play a key role as carriers of QS signaling within OBM

**Fig. 3:**
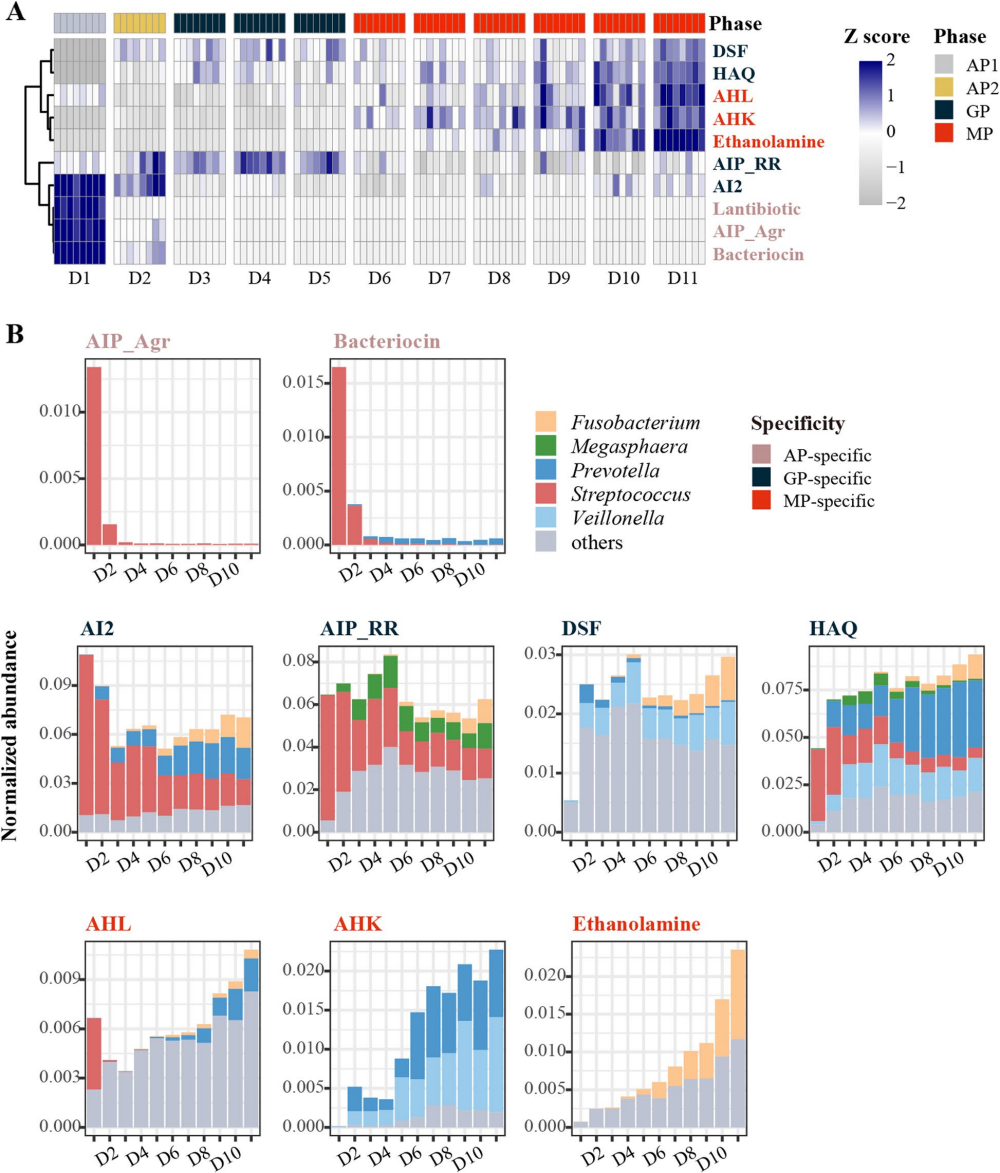
Time-series analysis of quorum sensing (QS) pathways retrieved from oral biofilm microbiota (OBM). **A** Sequential enrichment and classification of QS pathways into three types: Adapting Phase (AP)-, Growth Phase (GP)-, and Mature Phase (MP)-specific. The columns represent the samples that were collected daily throughout the OBM assembly process. Each column corresponds to one sample, and the specific collection day is indicated below each column. **B** The dominant genera that participate in QS signaling during AP, GP, and MP stages of OBM assembly

The QS pathways identified in this study were found to be sequentially enriched and converted during the AP-, GP-, and MP-assembling periods of OBM (Fig. S3). Specific QS pathways, such as those based on AIP_Agr-Fsr_Like, Lantibiotics, and Bacteriocin-II, were found to be AP-specific, with enrichment during AP but a sharp decrease once assembling into GP (Fig. 3A and B). The genus *Streptococcus* was the main participant involved in the AP-specific QS communications, utilizing these pathways to regulate adhesion [51], competence [49], biofilm formation [47], toxin production [57], and other related metabolisms involved in interference competition, preventing the invasion of other species, and establishing itself in a new community. The AP-QS communications enhanced the dominant colonization by *Streptococcus* to initiate the assembly of OBM. Similarly, MP-specific QS pathways, such as those based on AHK, AHL, and ethanolamine (Fig. 3A), were enriched during the MP period and mediated signal transmission among MP bacteria, especially *Prevotella* and *Fusobacterium*, and *Bacteroides* (Fig. 3B). MP-QS pathways regulate biofilm formation [72, 73], antibiotic resistance [75], virulence expression [74], and interspecies and even interkingdom cross-signaling [71, 73, 74], and stimulate the immune system of the host [76, 77], playing a crucial role in the formation of mature OBM.

The other four QS pathways based on AI-2, AIP_RRNPP, DSF, and HAQ were classified as GP-specific and had relatively stable abundances throughout the assembling process (Fig. 3B). GP-QS pathways have been reported to mediate interspecies cross-talk [60, 61, 66–69], facilitating collaborative multispecies biofilm formation (Table 1). More importantly, the GP-QS pathways undertook longitudinal transmission of QS signals from initial colonizers to late colonizers, thereby promoting steady assembly of OBM from AP to MP period.

### The longitudinal QS network shaped the direction of OBM assembly

**Fig. 4:**
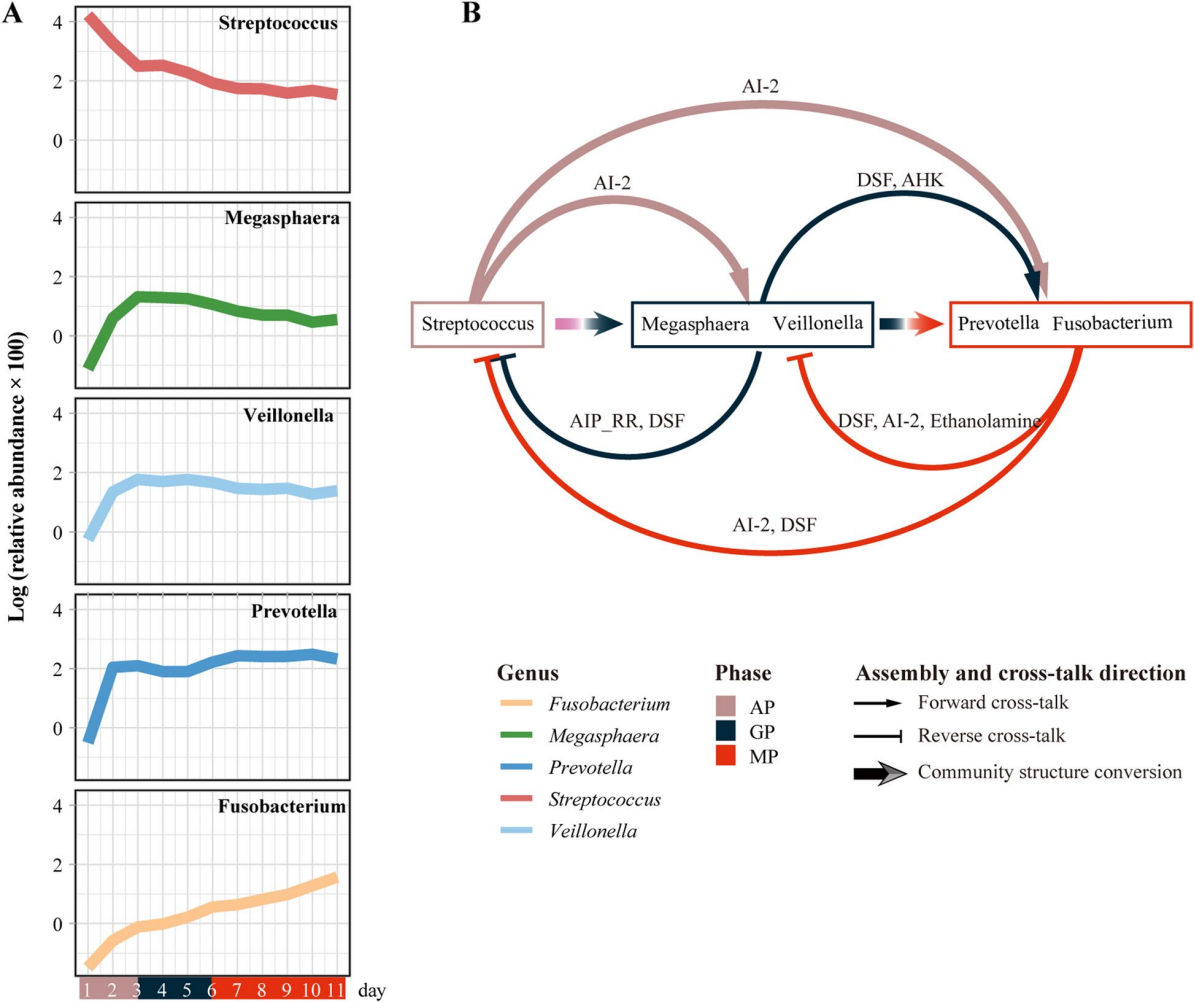
The impact of longitudinal quorum sensing (QS) on oral biofilm microbiota (OBM) assembly. **A** Proliferation trends of the five QS hubs during OBM assembly, with arrows indicating periods of cross-talk. **B** Bidirectional cross-talk among the QS hubs shaping the directional conversion of community structure. Forward cross-talk promoted the colonization and reproduction of later arrivals, while reverse cross-talk inhibited the further proliferation of former colonists

By doing BLASTP, we identified key QS signal synthesis and signal reception proteins involved in QS pathways and annotated them to respective species (Fig. 3, Figure S5 and Table 1). Species that possess QS signal synthase have the ability to produce and release QS signals, which are then sensed by other species that possess receptors. We refer to this type of interaction as “cross-talk”. The sequentially dominant *Streptococcus*, *Megasphaera* and *Veillonella*, *Prevotella*, and *Fusobacterium* were found to be responsible for multiple QS signals synthesizing and sensing during the assembly of OBM by possessing the related signal synthases and receptors. That means the interspecies cross-talk will be intensive among these core QS generalists. Two types of cross-talk were identified among the QS hubs: forward and reverse. The forward cross-talk delivered QS signal from the OBM of the previous period to the later period, such as the transmission of AI-2 from *Streptococcus* to *Megasphaera* and *Veillonella*. Conversely, the reverse type conveyed QS signal from the OBM of the later period to the former period, such as the transmission of AI-2 from *Fusobacterium* to previously colonized *Streptococcus* (Fig. 4B). Both types of cross-talk were expected to notify responders in time to cooperate with their corresponding signal synthesizers. However, the difference between them is that the forward cross-talk was found to be conducive to the colonization and reproduction of later arrivals, while the reverse type inhibited the further proliferation of former colonists (Fig. 4A). Overall, bidirectional cross-talk among QS hubs was identified as a key factor in shaping the directional transformation of bacterial structure during OBM assembly.

### AI-2 interfering experiment verified that the longitudinal QS network plays a crucial role in shaping OBM assembly

According to the longitudinal QS network, AI-2-based QS is the only one mediating both forward and reverse cross-talk across the whole assembling process (Fig. 4B). Therefore, AI-2 based subnetwork is more potentially involved in promoting OBM assembly than the others. If the delivery of AI-2 in OBM was blocked, according to our assumption, the proliferation of *Megasphaera* and *Veillonella* would be directly delayed, thereby delaying the conversion of downstream OBM; meanwhile, the reverse AI-2-based cross-talk would be deactivated, that is, AI-2 synthesized from *Prevotella*, *Fusobacterium* can no longer act backwards on initially colonized *Streptococcus*, resulting in a longer proliferation time and higher abundance of *Streptococcus* than those without AI-2 interference. This will also inhibit community conversion and normal assembly of OBM (Fig. S5).

**Fig. 5:**
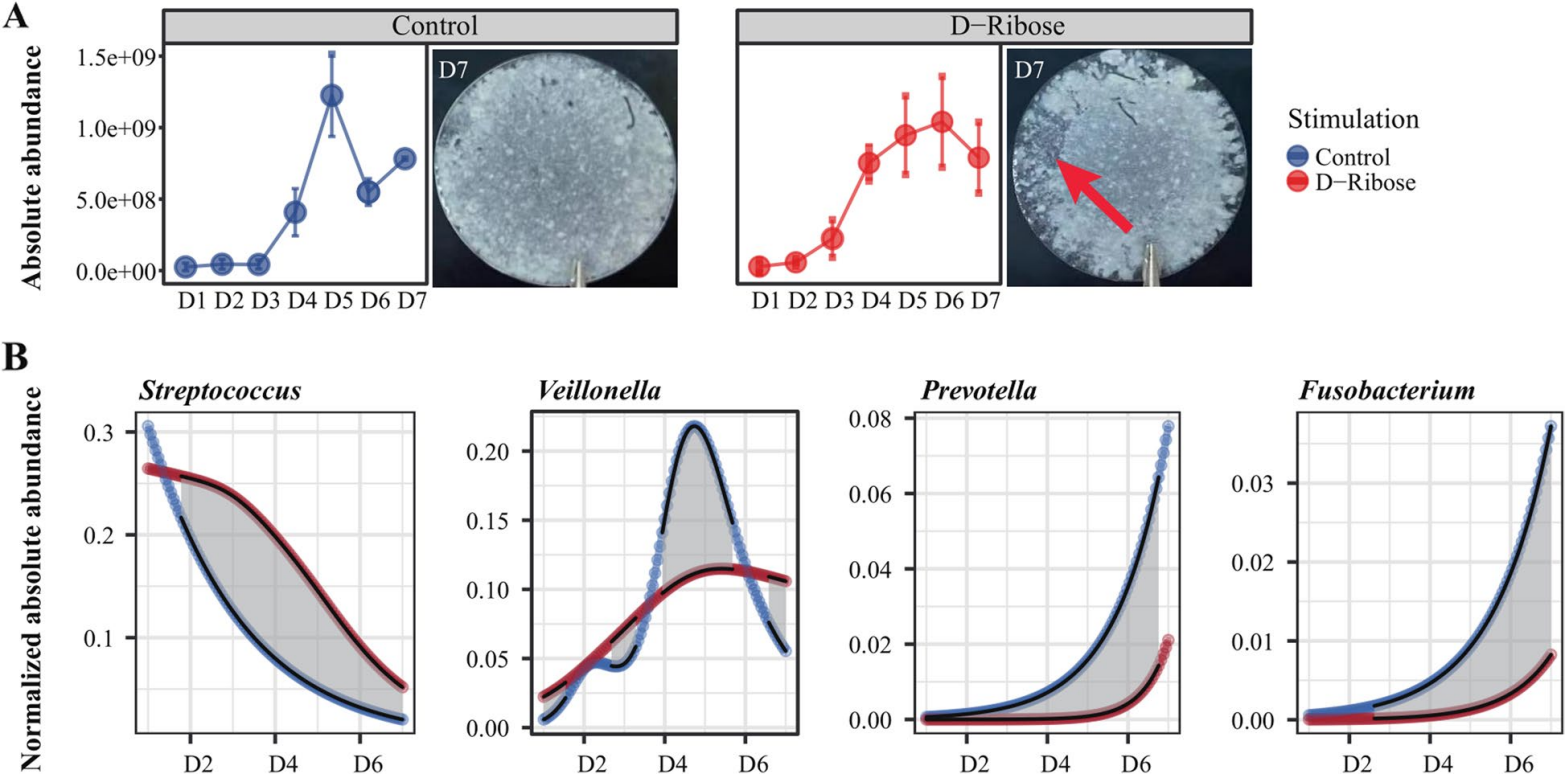
Validation of the driving role of the AI-2-based quorum sensing (QS) subnetwork in OBM assembly. **A** Biomass measurements of OBM in control and AI-2 interference experiments, as well as photographs at day 7 of growth. **B** Longitudinal differential analysis using MetaLonDA revealed temporal changes in normalized absolute abundances of core QS hubs between control and AI-2 interfering groups, with the gray shaded area indicating the significant time interval during which differences were observed

To verify our hypothesis, D-ribose, a frequently used substrate competition inhibitor of AI-2, was supplied to deactivate AI-2 singling during OBM assembling (Fig. S2). We then analyzed changes in OBM biomass, growth rate of key QS hubs to evaluate the power of AI-2 subnetwork in shaping OBM assembly. The results turned out that OBM developed in the AI-2 interference group was more fragile compared to the control (Fig. 5A). Specifically, time needed for conversion of OBM from GP to MP was 1 day delayed when AI-2 signal was interfered (Fig. 5A), which was coincident with delayed proliferation of *Veillonella* (Fig. 5B). Moreover, *Streptococcus* proliferation was enhanced in AI-2 interference group (Fig. 5B). Its maximum biomass doubled, and its proliferation was prolonged compared with the control group (Fig. 5B, Fig. S6). In the meantime, growth of downstream QS hubs *Prevotella* and *Fusobacterium* were suppressed with significant lower biomass than the control group (Fig. 5B, Fig. S6). It can be seen that AI-2-based QS controlled the turnover of OBM community structure via timely regulation of the growth rate of key QS hubs, thereby shaping OBM assembly.

## Discussion

In this study, we have successfully developed an optimized in vitro oral biofilm model (OBMA) by incorporating human saliva and an optimized growth medium. This model not only demonstrates greater diversity but also provides growth conditions that closely resemble a real oral biofilm, thus yielding more realistic and reliable outcomes compared to previous in vitro models. By mixing saliva from multiple individuals, the aim is to create an environment conducive to studying the common characteristics of oral biofilm microbiota within hosts, while still accounting for individual specificity in the oral microbiome. Additionally, our findings from cohort studies provide supporting evidence for the reliability of reconstructing human oral biofilm using the OBMA model. Specifically, both the oral biofilms developed in the human oral cavity and in our OBMA model exhibited similar proliferation patterns, showing a rapid proliferation phase followed by reaching a stable state [78]. Secondly, a consistent pattern was observed during the transition of microbial community structures. Specifically, the initial population of human oral microbiome is facultative anaerobic Streptococcus and was ultimately replaced by gram-negative anaerobic *Fusobacteria*, *Prevotella*, and *Porphyromonas* along with the formation of oral biofilm [3, 78]. Additionally, the core genus *Veillonella* observed in the growing period in this study has been confirmed as a bridging species critical in guiding the development of multispecies biofilms in the human oral microenvironment [79, 80]. Above all, our constructed OBMA model is reliable to restore the maturation trajectory of human oral biofilm. The OBMA model developed in this study not only holds potential for exploring the regulatory mechanisms of oral biofilm assembly but also can be utilized in early-stage clinical research for screening and evaluating the effectiveness of drugs in controlling oral biofilms.

Our study explores the regulatory role of bacterial QS in the assembly of OBM regardless of host factors. Using the OBMA model, we can observe the entire assembly process while consuming fewer resources and manpower compared to cohort studies.

In contrast to previous strategies based on metabolic collaboration and competition, our study suggests that QS-based interactions regulate microbiota assembly ahead of the onset of other forms of metabolic interactions. QS signals are released and accumulated in the logarithmic growth phase of bacteria to mediate interspecific interactions before most enzymes and metabolites synthesized in stationary growth phase. These signals recruit bacteria clustering with kinship to facilitate microbiota assembly [81]. QS is also responsible for biofilm matrix formation, affecting the microbiota’s structure and function. QS is the fundamental underlying mechanism regulating microbiota assembly.

While it has been suggested QS can promote or inhibit biofilm formation of monocultures, it is challenging to predict how QS plays a role in the assembly of a complex microbial community with hundreds of species based solely on monoculture test results. Our study uncovers the successions of community structure and QS pathways during OBM assembly. We constructed a longitudinal QS network based on this and find it possessing more diverse and complex QS pathways than ever reported. These pathways sequentially enrich and play roles in initial colonizing, exponential proliferation, and maturation, respectively. QS signals in OBM transmit bidirectionally, allowing key QS hubs to regulate transformation of community structure to drive OBM assembly. The construction and regulatory effects of the QS network in the assembly of complex microbiota are unprecedented. Our study provides a new perspective for exploring the underlying mechanism of flora assembly.

The longitudinal QS network proposed in our study provides new possibilities for the application of QS regulation mechanisms in the manipulation of complex microbiota. Based on this network, we accurately predicted and verified the reverse effects of the forward and reverse AI-2 signaling and their respective target QS hubs. Our research results suggest that the exploration of QS regulation can be extended to other human microbiota research, such as the evolution and assembly of colorectal cancer microbiota. By targeting the core QS signals and QS hubs, we can regulate the proliferation of microbiota members, reshape the microbiota in a targeted manner, and ultimately develop new strategies to prevent or treat microbiota-related diseases.

## Conclusion

Our results establish a longitudinal QS network scaffolded by dominant bacteria in shaping OBM assembly. This network demonstrates the predictability and controllability of QS interference in regulating the microbiota, thereby expanding our understanding of the mechanism governing microflora assembly, and boosting confidence in applying QS networks to modulate assembly and development of human microbiota.

### Supplementary Information

**Additional file 1: Fig. S1.** Workflow for retrieval of QS proteins from OBM metagen. **Fig. S2.** The study design of QS-interfering experiment. **Fig. S3.** Profiles of QS pathways of OBM shown in robust principal component analysis (PCA) biplot, whose transition correlated with the turn over of the assembly phase of OBM. **Fig. S4.** The dominant genera that participate in QS signal receptions during AP, GP, and MP stages of OBM assembly. **Fig. S5.** Diagram of dynamical responses of key QS hubs in the AI-2 interfering experiment. A) Expected proliferation trends of the five QS hubs during OBM assembly when AI-2 signaling gets interfered. B) Diagram of changes in the bidirectional cross talk among the QS hubs when AI-2 signaling gets interfered. **Fig. S6.** Validation of driving role by AI-2 based QS subnetwork in the assembly of OBM. Longitudinal differential analysis using MetaLonDA revealed temporal changes in absolute abundances of core QS hubs between control and AI-2 interfering groups, with the gray shaded area indicating the significant time interval during which differences were observed.

**Additional file 2: Table S1.** Proposed roles for the homologs of reference QS proteins found within the oral biofilm microbiota.

## Data Availability

The sequencing data of metagenome used in this study have been deposited in the GenBank Short Read Archive with accession number PRJNA983519. The accession number can be found below: http://www.ncbi.nlm.nih.gov/sra. The scripts for the retrieval of QS proteins from OBM metagenomes are available in the following Git repository: https://github.com/TaoDing/QS_01.

## Abbreviations

QS: Quorum sensing
OBM: Oral biofilm microbiota
OBMA: Oral biofilms microbiota assembling
AIP: Autoinducing peptide
AI-2: Autoinducer-2
OGM: Optimized growth medium
HA: Hydroxyapatite
qPCR: Quantitative PCR
CDD: Conserved domain database
AP: Adapting phase
GP: Growing phase
MP: Mature phase
DSF: Diffusible signal factors
HAQ: 4-Hydroxy-2-alkylquinoline
AHK: α-Hydroxyketones
