# Supplementary material for: Bacterial quorum sensing orchestrates longitudinal interactions to shape microbiota assembly
Source: Microbiome. 2023 Nov 6;11:241. doi: 10.1186/s40168-023-01699-4 (PMC10626739; doi:10.1186/s40168-023-01699-4)
Supplement: Supplementary file 2 — Additional file 1: Fig. S1. Workflow for retrieval of QS proteins from OBM metagen. Fig. S2. The study design of QS-interfering experiment. Fig. S3. Profiles of QS pathways of OBM shown in robust principal component analysis (PCA) biplot, whose transition correlated with the turn over of the assembly phase of OBM. Fig. S4. The dominant genera that participate in QS signal receptions during AP, GP, and MP stages of OBM assembly. Fig. S5. Diagram of dynamical responses of key QS hubs in the AI-2 interfering experiment. A) Expected proliferation trends of the five QS hubs during OBM assembly when AI-2 signaling gets interfered. B) Diagram of changes in the bidirectional cross talk among the QS hubs when AI-2 signaling gets interfered. Fig. S6. Validation of driving role by AI-2 based QS subnetwork in the assembly of OBM. Longitudinal differential analysis using MetaLonDA revealed temporal changes in absolute abundances of core QS hubs between control and AI-2 interfering groups, with the gray shaded area indicating the significant time interval during which differences were observed. [file 40168_2023_1699_MOESM1_ESM.pdf]

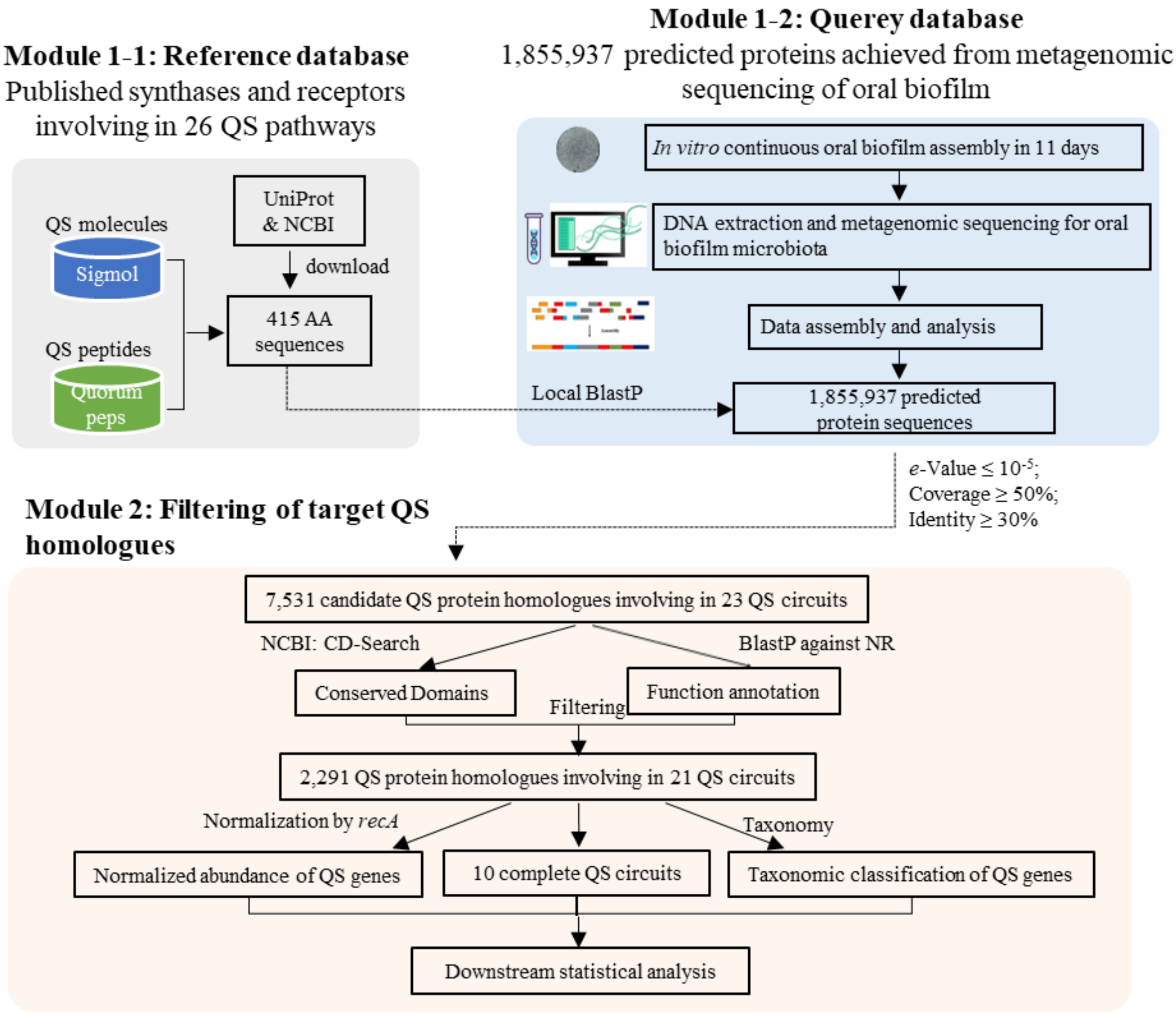

Fig. S1 Workflow for retrieval of QS proteins from OBM metagenomes

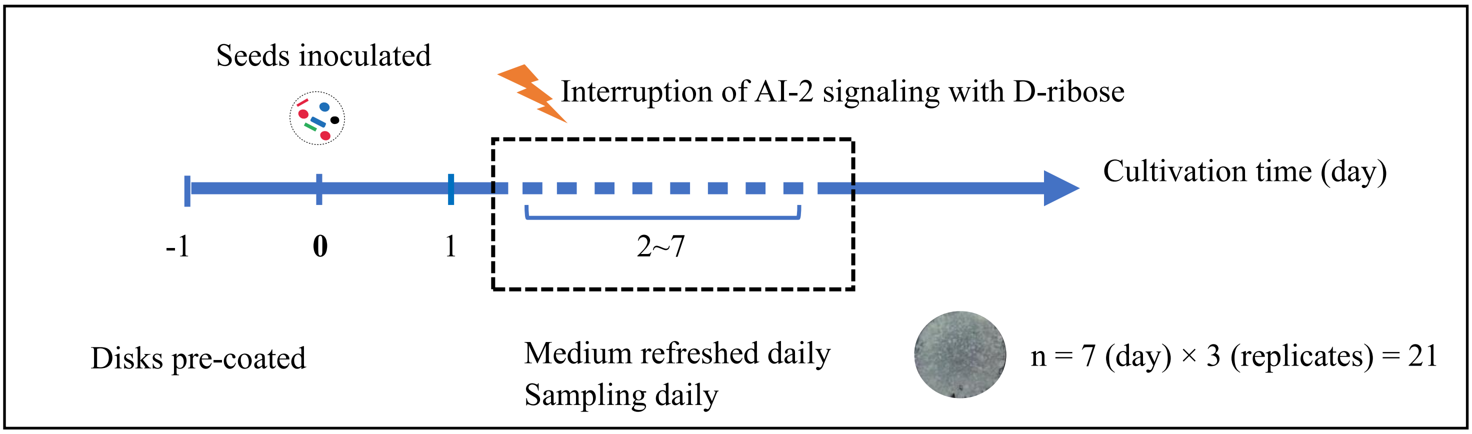

Fig. S2 The study design of QS-interfering experiment.

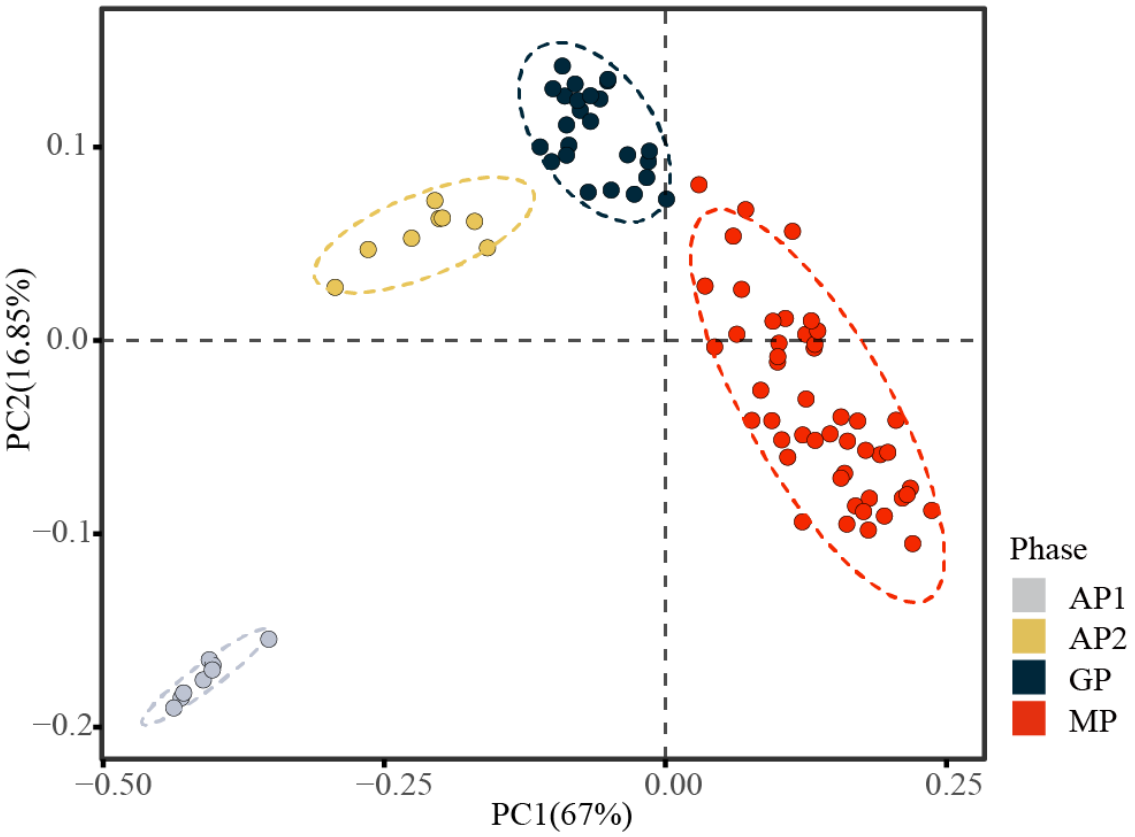

Fig. S3 Profiles of QS pathways of OBM shown in robust principal component analysis (PCA) biplot, whose transition correlated with the turn over of the assembly phase of OBM.

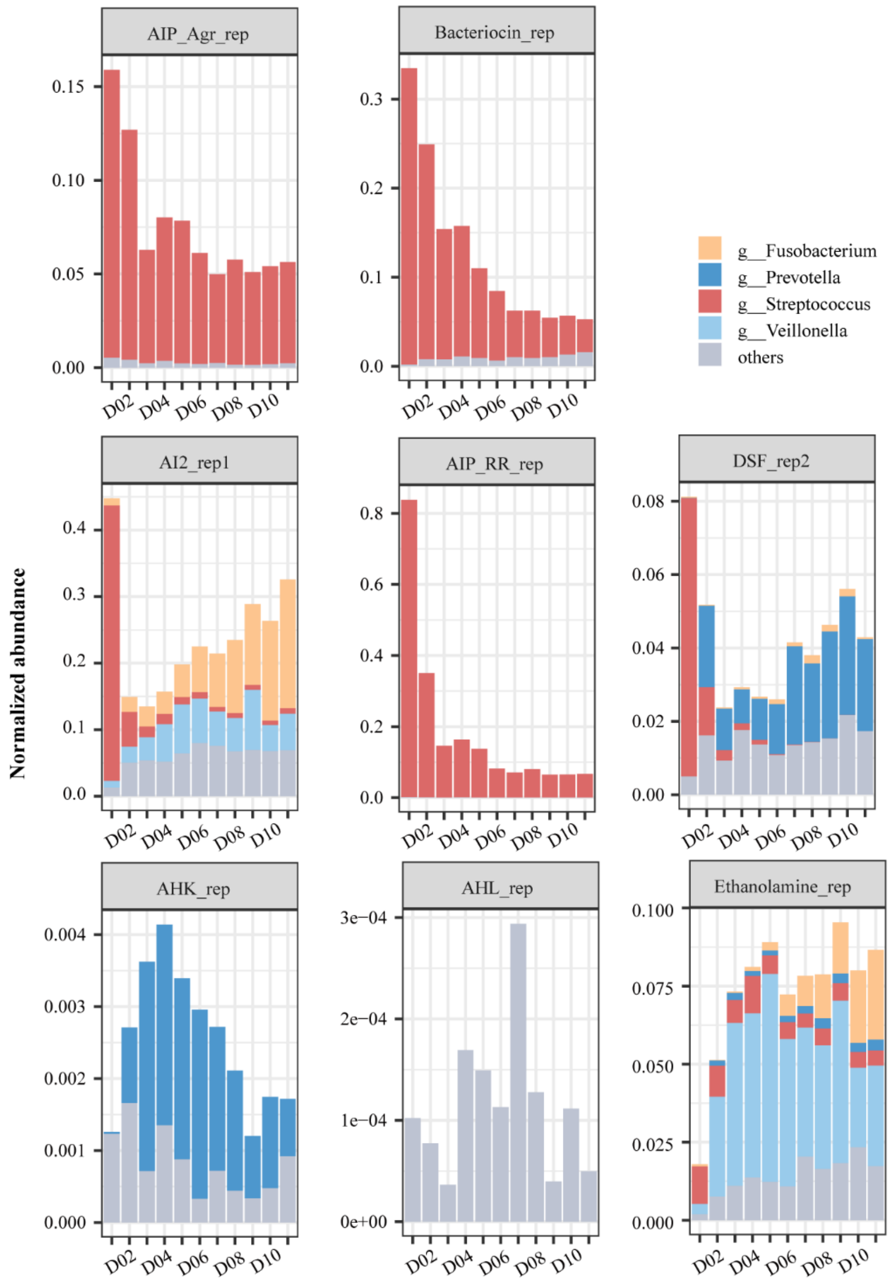

Fig. S4 The dominant genera that participate in QS signal receptions during AP, GP, and MP stages of OBM assembly.

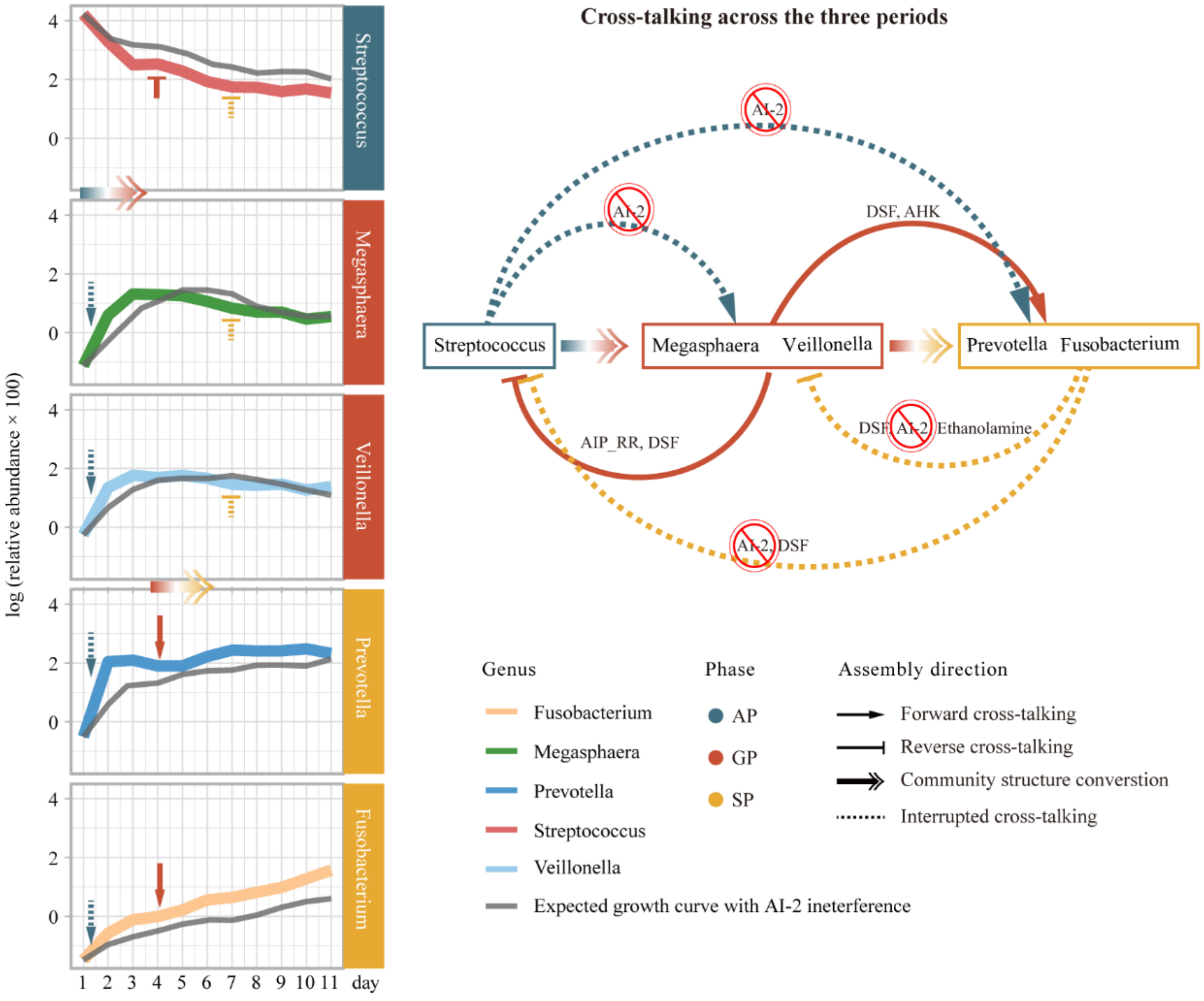

Fig. S5 Diagram of dynamical responses of key QS hubs in the AI-2 interfering experiment. A) Expected proliferation trends of the five QS hubs during OBM assembly when AI-2 signaling gets interfered. B) Diagram of changes in the bidirectional cross-talk among the QS hubs when AI-2 signaling gets interfered.

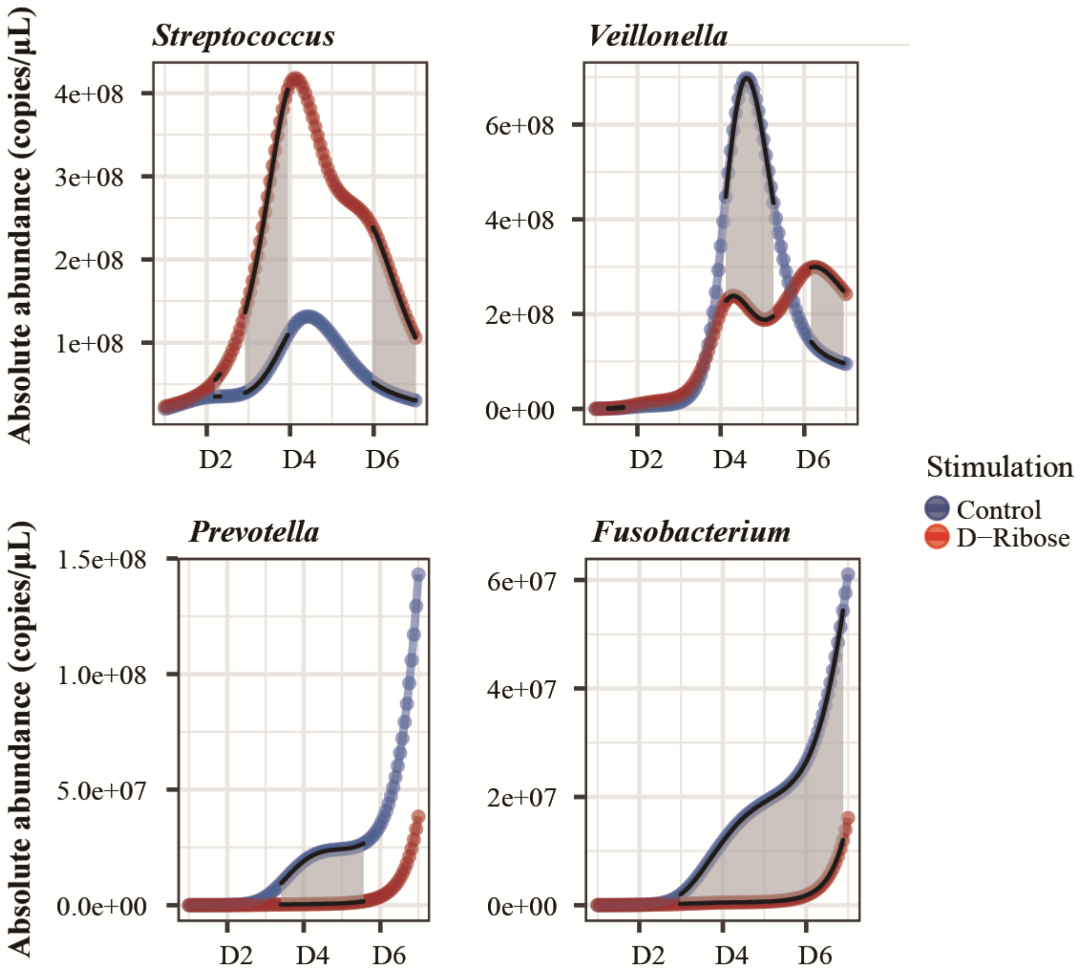

Fig. S6 Validation of driving role by AI-2 based QS subnetwork in the assembly of OBM. Longitudinal differential analysis using MetaLonDA revealed temporal changes in absolute abundances of core QS hubs between control and AI-2 interfering groups, with the gray shaded area indicating the significant time interval during which differences were observed.
