# Supplementary material for: Bacterial quorum sensing orchestrates longitudinal interactions to shape microbiota assembly
Source: Microbiome. 2023 Nov 6;11:241. doi: 10.1186/s40168-023-01699-4 (PMC10626739; doi:10.1186/s40168-023-01699-4)
Supplement: Supplementary file 3 — Additional file 2: Table S1. Proposed roles for the homologs of reference QS proteins found within the oral biofilm microbiota. [file 40168_2023_1699_MOESM2_ESM.docx]

**Table S1. Proposed roles for the homologs of reference QS proteins found within the oral biofilm microbiota**

| **Signal** | **Reference protein** | **Mapped genus** | | | **Possible roles regulated by the QS circuit** |
| --- | --- | --- | --- | --- | --- |
|  |  | **AP1** | **GP** | **MP** |  |
| **AP specific** | | | | | |
| Photopyrone | PpyS synthase  (Photorhabdus thracensis) | Streptococcus  Rothia | \ | \ | Cell–cell communication as in Photorhabdus (Brameyer and Heermann, 2016) |
| TDA | TdaD; TdaE; TdaF synthase  (Phaeobacter inhibens DSM 17395) | Streptococcus  Latilactobacillus | Streptococcus  Veillonella | Veillonella  Bacteroides  Fusobacterium | Colonization success against other bacteria (Brinkhoff et al., 2004; Porsby et al., 2008);  Autoinducer of its own synthesis (Geng and Belas, 2010) |
| **GP specific** | | | | | |
| Butyolactone | ArpA receptor  (one component) | Streptococcus | Streptococcus  Lysinibacillus  Clostridium | Streptococcus  Lysinibacillus  Clostridium  Unclassified Lachnospiraceae  Dialister | Eavesdropping on host QS molecules (Polkade et al., 2016) |
| AI-3 | QseC receptor  (two component)  (Escherichia coli) | Streptococcus | Streptococcus  Ruminococcus  Actinomyces  Bacillus  Prevotella | Ruminococcus  Unclassified Lachnospiraceae  Prevotella | Mediating inter-kingdom sensing by responding to the host-derived adrenergic signals epinephrine and norepinephrine (Clarke et al., 2006) |
| DPO | Tdh synthase  (Vibrio cholerae) | Streptococcus  Flaviflexus  Bifidobacterium | Streptococcus  Flaviflexus  Bifidobacterium | Streptococcus  Oscillibacter | Regulating virulence and biofilm formation as in Vibrios (Huang et al., 2020) |
| IQS | AmbB synthase | Streptococcus | Streptococcus  Unclassified Eubacteriales Family  Prevotella | ~~Streptococcu~~s  Unclassified Eubacteriales Family  Prevotella | Intercellular communication, integrating the QS network, stress response and bacterial virulence (Lee et al., 2013) |
| AIP_Eukaryota | Tup1 receptor | Pauljensenia | Pauljensenia  Actinomyces  Paraglomus  Dentiscutata  Serendipita | Pauljensenia  Actinomyces | Density-dependent growth and cell-to-cell communication exists in the Kingdom Fungi (Lee et al., 2007) |
| **MP specific** | | | | | |
| DKP | PhcE; PhcF synthase  (Pseudomonas aeruginosa PAO1) | \ | Lysinibacillus  Peptoniphilus  Desulgitobacterium | Lysinibacillus  Peptoniphilus  Desulgitobacterium  mediterraneibacter | Promoting biofilm formation, spoilage capacity (Wang et al., 2019) and production of TVB-N ( total volatile basic nitrogen), TMA, putrescine and extracellular proteases (Gu et al., 2013) |
| DAR\CHD | DarB synthase  (Photorhabdus asymbiotica) | Actinobacillus | Prevotella | Prevotella  Pseudomonas  Tannerella | Cell clumping (Brameyer et al., 2015) |
| Indole | TnaA synthase (Vibrio) | \ | Odoribacter  Peptoniphilus | Rhodopseudomonas  Prevotella  Odoribacter  Peptoniphilus  Rhodopseudomonas  Unclassified Lachnospiraceae | Modulation of biofilm formation, motility and antibiotic resistance within microbial communities (Kim and Park, 2015)  Inhibition of QS receptors in other prokaryotes (Kim and Park, 2013) |
| Methyl Esther | PhcS receptor  (two component) | Neisseria | Prevotella  Schaalia  Sulfurospirillum | Veillonella  Wolinella | Production of EPS and other virulence factors (Flavier et al., 1997; Kai, 2018)  Regulating bacteria−host interactions (Kai, 2018) |

Brameyer, S. and Heermann, R., 2016. Quorum sensing and LuxR solos in Photorhabdus, The molecular biology of Photorhabdus bacteria. Springer, pp. 103-119.

Brameyer, S., Kresovic, D., Bode, H.B. and Heermann, R., 2015. Dialkylresorcinols as bacterial signaling molecules. Proceedings of the National Academy of Sciences, 112(2): 572-577.

Brinkhoff, T. et al., 2004. Antibiotic production by a Roseobacter clade-affiliated species from the German Wadden Sea and its antagonistic effects on indigenous isolates. Applied environmental microbiology, 70(4): 2560-2565.

Clarke, M.B., Hughes, D.T., Zhu, C., Boedeker, E.C. and Sperandio, V., 2006. The QseC sensor kinase: a bacterial adrenergic receptor. Proceedings of the National Academy of Sciences, 103(27): 10420-10425.

Flavier, A.B., Clough, S.J., Schell, M.A. and Denny, T.P., 1997. Identification of 3‐hydroxypalmitic acid methyl ester as a novel autoregulator controlling virulence in Ralstonia solanacearum. Molecular microbiology, 26(2): 251-259.

Geng, H. and Belas, R., 2010. Expression of tropodithietic acid biosynthesis is controlled by a novel autoinducer. Journal of bacteriology, 192(17): 4377-4387.

Gu, Q., Fu, L., Wang, Y. and Lin, J., 2013. Identification and characterization of extracellular cyclic dipeptides as quorum-sensing signal molecules from Shewanella baltica, the specific spoilage organism of Pseudosciaena crocea during 4 C storage. Journal of agricultural food chemistry, 61(47): 11645-11652.

Huang, X. et al., 2020. Mechanism underlying autoinducer recognition in the Vibrio cholerae DPO-VqmA quorum-sensing pathway. Journal of Biological Chemistry, 295(10): 2916-2931.

Kai, K., 2018. Bacterial quorum sensing in symbiotic and pathogenic relationships with hosts. Bioscience, Biotechnology, and Biochemistry, 82(3): 363-371.

Kim, J. and Park, W., 2013. Indole inhibits bacterial quorum sensing signal transmission by interfering with quorum sensing regulator folding. Microbiology, 159(Pt_12): 2616-2625.

Kim, J. and Park, W., 2015. Indole: a signaling molecule or a mere metabolic byproduct that alters bacterial physiology at a high concentration? Journal of Microbiology, 53(7): 421-428.

Lee, H., Chang, Y.C., Nardone, G. and Kwon‐Chung, K.J., 2007. TUP1 disruption in Cryptococcus neoformans uncovers a peptide‐mediated density‐dependent growth phenomenon that mimics quorum sensing. Molecular microbiology, 64(3): 591-601.

Lee, J. et al., 2013. A cell-cell communication signal integrates quorum sensing and stress response. Nature chemical biology, 9(5): 339-343.

Polkade, A.V., Mantri, S.S., Patwekar, U.J. and Jangid, K., 2016. Quorum sensing: an under-explored phenomenon in the phylum Actinobacteria. Frontiers in microbiology, 7: 131.

Porsby, C.H., Nielsen, K.F. and Gram, L., 2008. Phaeobacter and Ruegeria species of the Roseobacter clade colonize separate niches in a Danish turbot (Scophthalmus maximus)-rearing farm and antagonize Vibrio anguillarum under different growth conditions. Applied environmental microbiology, 74(23): 7356-7364.

Wang, Y., Wang, F., Wang, C., Li, X. and Fu, L., 2019. Positive regulation of spoilage potential and biofilm formation in Shewanella baltica OS155 via quorum sensing system composed of DKP and orphan LuxRs. Frontiers in microbiology, 10: 135.
